# The use of opioids in low acuity pediatric trauma patients

**DOI:** 10.1371/journal.pone.0226433

**Published:** 2019-12-16

**Authors:** Ashley A. Foster, John J. Porter, Florence T. Bourgeois, Rebekah Mannix

**Affiliations:** 1 Division of Emergency Medicine, Boston Children’s Hospital, Boston, Massachusetts, United States of America; 2 Departments of Pediatrics and Emergency Medicine, Harvard Medical School, Boston, Massachusetts, United States of America; 3 Computational Health Informatics Program, Boston Children’s Hospital, Boston, Massachusetts, United States of America; Medical University Graz, AUSTRIA

## Abstract

**Objective:**

To describe temporal trends and factors associated with opioid administration among children discharged from the emergency department (ED) after a trauma visit.

**Methods:**

This was a cross-sectional study of ED visits for children <19 years old who received a trauma-related diagnosis and were discharged from the ED. Data were obtained from the National Hospital Ambulatory Medical Care Survey 2006–2015.

**Outcome measures:**

Administration of an opioid medication either during the ED visit or as a discharge prescription. Survey-adjusted regression analyses were used to determine the probability of a patient receiving an opioid medication.

**Results:**

During the study period, there were 19,241 pediatric trauma visits discharged from the ED, of which 14% were associated with an opioid. Opioid administration decreased by nearly 30% during the study period (p<0.001 for trend). In multivariable analysis, patient factors associated with opioid administration were adolescent age, evening visit, region of the country, and severe pain score. The diagnosis associated with the most opioids was ankle sprain and the diagnosis with the highest rate of opioid administration was radius fracture. The most common opioid administered to children under 12 years of age was acetaminophen-codeine.

**Conclusions:**

Opioid administration appears to be decreasing among pediatric patients presenting to the ED with trauma, but a high number of children continue to be exposed to opioids every year. Further education on opioid sparing pain management strategies may be warranted to decrease opioid exposure, including the inappropriate use of codeine, in this low risk trauma population.

## Introduction

The opioid crisis in the United States (US) affects people of all ages, including children and adolescents. Much of the literature on opioid use has focused on the adult patient population. However, adolescents are especially vulnerable to misuse of prescription medications.[1] Even short-term prescriptions are associated with misuse for certain youth.[2] Additionally, from 2012–2016, unintentional deaths from poisoning, including opioid-related deaths, became the leading cause of unintentional injury death in those 15–34 years of age.[3] Taken together, these studies suggest that efforts aimed at minimizing opioid exposures among children may have important public health implications.

The emergency department (ED) may contribute to opioid exposures in children and adolescents and opioid use for pain-related pediatric ED visits increased from 2001–2010.[4] Though a prior study showed that EDs do not contribute substantially to the high rates of opioid prescriptions, this study excluded children.[5] In fact, little is known about opioid administration and prescribing patterns for children receiving care in EDs.

Trauma related complaints are a common presentation for children and adolescents seeking evaluation in the ED, with one recent study reporting trauma as representing approximately 26% of all pediatric ED visits—the most frequent reason for visits in this age group.[6] Injury and trauma-related complaints are often treated with opioid medications, although there is increasing evidence supporting the use of nonsteroidal anti-inflammatory medications for these injuries.[7–9]

A prior study demonstrated that pediatric ED visits associated with a bone fracture had the highest rate of opioid prescribing.[10] Understanding practice patterns around pain management in such visits, which are high risk for receiving opioids are necessary prior to interventions targeting reductions in opioid exposures. In this study, we explore rates and characteristics of opioid administration among pediatric patients discharged from the ED after a trauma-related visit, including temporal trends over a 10-year period.

## Methods

### Study design

This study was approved by the Institutional Review Board of Boston Children’s Hospital. It is a cross sectional study of the data collected in the National Hospital Ambulatory Medical Care Survey (NHAMCS) from 2006–2015. The NHAMCS is an annual, national probability sample of ambulatory visits made to non-federal, general and short-stay hospitals in the US conducted by the Centers for Disease Control and Prevention at the National Center for Health Statistics (NCHS).[11] National estimates are derived from data collected on approximately 25,000 visits annually from approximately 600 hospital EDs. Details of NHAMCS methods are published extensively elsewhere.[12]

### Data source and study population

We identified visits for children <19 years old who received a trauma-related diagnosis and were discharged from the ED. Trauma visits were identified based on any ICD-9 codes listed for patient visit corresponding to 800–904, 910–959 (800–804 fracture of skull, 805–809 fracture of spine and trunk, 810–819 fracture of upper limb, 820–829 fracture of lower limb, 830–839 dislocation, 840–848 sprains and strains of joints and adjacent muscles, 850–854 intracranial injury excluding those with skull fracture, 860–869 internal injury of chest abdomen and pelvis, 870–879 open wound of head neck and trunk, 880–887 open wound of upper limb, 890–897 open wound of lower limb, 900–904 injury to blood vessels, 910–919 superficial injury, 920–924 contusion with intact skin surface, 925–929 crushing injury, 930–939 effects of foreign body entering through orifice, 940–949 burns, 950–957 injury to nerves and spinal cord, 958–959 certain traumatic complications and unspecified injuries). The population was limited to discharged patients to capture lower acuity injuries stable for discharge from the ED.

### Measurement and outcome

Our outcome measure was administration of an opioid medication (during the ED visit, as a discharge prescription or both). We included both ED administration of opioids and discharge prescription of opioids in our outcome definition as opioid sparing practices have been advocated in recent consensus statements for both acute ED pain management and discharge prescriptions.[13,14] We defined an opioid medication as any scheduled medication regulated by the DEA, classified based on Multum’s lexicon Drug Database, codes 060 (narcotic analgesic) and 191 (narcotic analgesic combination).

For visits associated with an opioid, we examined the following patient and provider level variables: injury intent (unintentional, intentional, unknown), pain severity, injury severity score, time of day, discharge diagnoses, patient demographics (age, sex, race ethnicity), patient insurance type (private, not private), day of visit (weekday, weekend), provider type (resident physician, nurse practitioner, physician assistant). Intentional injury is defined by NHAMCS as “the injury/trauma or overdose/poisoning resulted from an act carried out on purpose by one or more persons with the intent of causing harm, injury, or death to another person”. Unintentional injury is defined as “The injury/trauma or overdose/poisoning was not purposefully inflicted with the intent to harm”. Unknown intent is defined as “The medical record clearly states that there is difficulty determining whether the event was intentional or accidental”.[15] Pain severity of the patient was assessed using a numerical scale ranging from 0 (no pain) to 10 (worst pain imaginable). [15] If a pediatric pain scale was used, consisting of 6 faces, the scale was adapted to the numerical scale by multiplying the value of the pediatric scale by two (e.g. for 2 on the faces scale, enter 4 and for 0 on faces scale enter 0).[16] For our analysis, we defined a score of 0 as “no pain”, 1 to 3 as “mild pain”, 4 to 6 as “moderate pain”, and 7 to 10 as “severe pain”. In NHAMCS, patient race (white, non-white) and ethnicity (Hispanic, non-Hispanic) are determined based on the observations of hospital personnel, unless it is hospital policy to ask patients directly for this information. The NHAMCS data do not include any direct measure of socioeconomic status, thus “type of insurance” serves as a surrogate measure, comparing private insurance to non-private insurance (combining the categories ‘Medicaid’, ‘self-pay,’ ‘no charge,’ and ‘other,’ and the categories ‘Medicare’ and ‘Workman's Compensation’). Hospital level variables included whether the ED was based in a pediatric or teaching hospital. We classified EDs in which 75% or more of all visits are for patients 18 years or younger as pediatric facilities.[17] Academic hospitals were defined as facilities in which at least 25% of the patients are evaluated by a resident physician.[18] Other hospital characteristics, included region (Northeast, South, Midwest, West), and hospital ownership (private, nonprofit, government). In addition, due to the US Food and Drug Administration and World Health Organization recommendations against utilizing codeine in children under 12 years of age, we ascertained the frequency of codeine administration in the ED and/or prescription for children < 12 years of age in our study population.[19] To account for injury severity, we used a previously validated diagnosis-based severity classification system.[20]

### Statistical analysis

Data were analyzed using the sampled visit weight that is the product of the corresponding sampling fractions at each stage in the sample design. The sampling weights have been adjusted by NCHS for survey nonresponse within time of year, geographic region, urban/rural and ownership designations, yielding an unbiased national estimate of ED visit occurrences, percentages, and characteristics. When generating estimates from survey data, we considered the survey design by specifying the primary sampling units and the patient visit sampling weights provided by NHAMCS (denoting the inverse of the probability that the observation is included), to ensure accurate estimates.[21]

We calculated descriptive statistics for the primary outcome, using the svy commands available in STATA to derive national estimates. All estimates are reported as rounded to the nearest 1,000.

Next, we performed bivariable logistic regression to identify the strength of association of opioid receipt and patient, provider, and hospital specific characteristics. We then performed multivariable logistic regression to adjust for confounding by specified covariates for which there was clinical or epidemiological plausibility. Variables with p< 0.10 in any of the bivariable analyses and variables that were a priori felt to have a potential relationship with overall opioid administration (gender, pediatric ED, academic ED, nurse practitioner present) were retained in our multivariable model. Significance in the final model was defined as a p-value of less than 0.05. Unless otherwise noted, percentages are expressed as survey-weighted proportions and all p-values are two-sided. All analyses were performed in STATA Version 15 (StataCorp, College Station, TX) using the suite of estimation commands for survey data (svyset and svy).

For missing data, four items were imputed by NHAMCS in every year of the study: patient’s age, sex, race, and ethnicity. On average, age, sex, race, and ethnicity were imputed for 0.71%, 0.73%, 14.0%, and 21.7% of records, respectively. Prior to 2012, triage level was also imputed at an average of 9.7%. For multivariate analyses, records with missing data on severity score (1.3%), injury intent (8.1%), and pain level (25.5%) were excluded from the analysis. Missingness on the pain level score was not significantly associated with any demographic factor except age, where the proportion of cases with missing pain scores was greater in children under the age of 5 compared to the older age groups.

## Results

**Fig 1:**
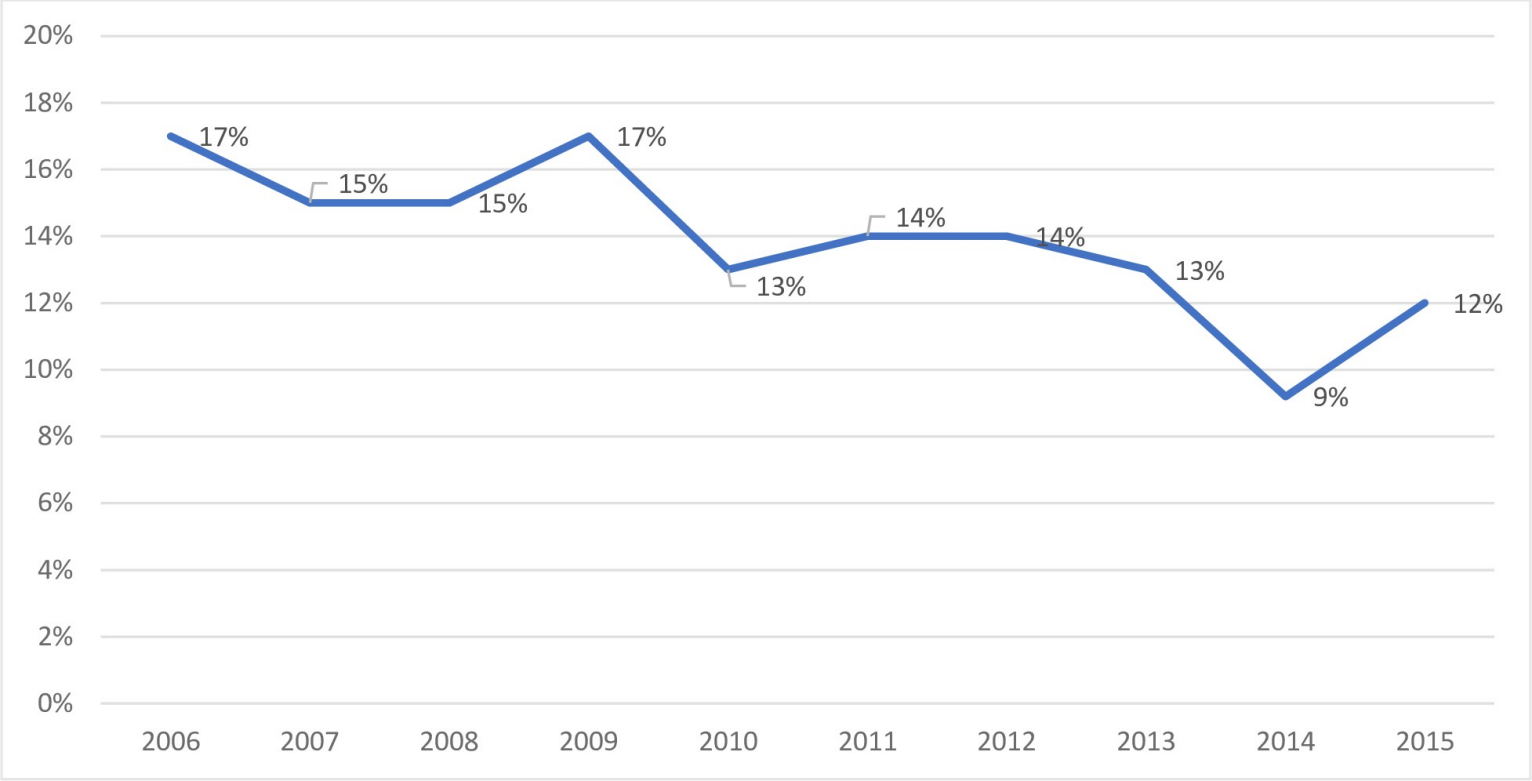
Percentage of patients given opioids by year.

During the ten-year study period, NHAMCS included data on 19,241visits to EDs in the US by patients under the age of 19 who were discharged from the ED with a trauma diagnosis. This represents an estimated 81 million visits. Among these visits, 2,608 (14%) representing 11 million visits in the US, were associated with an opioid medication. Of these, 72% received an opioid prescription at discharge. Over the study period, the proportion of visits associated with an opioid medication decreased by 29% (p-value <0.001), with a rate as high as 17% in 2006 decreasing to 12% in 2015 (Fig 1).

**Fig 2:**
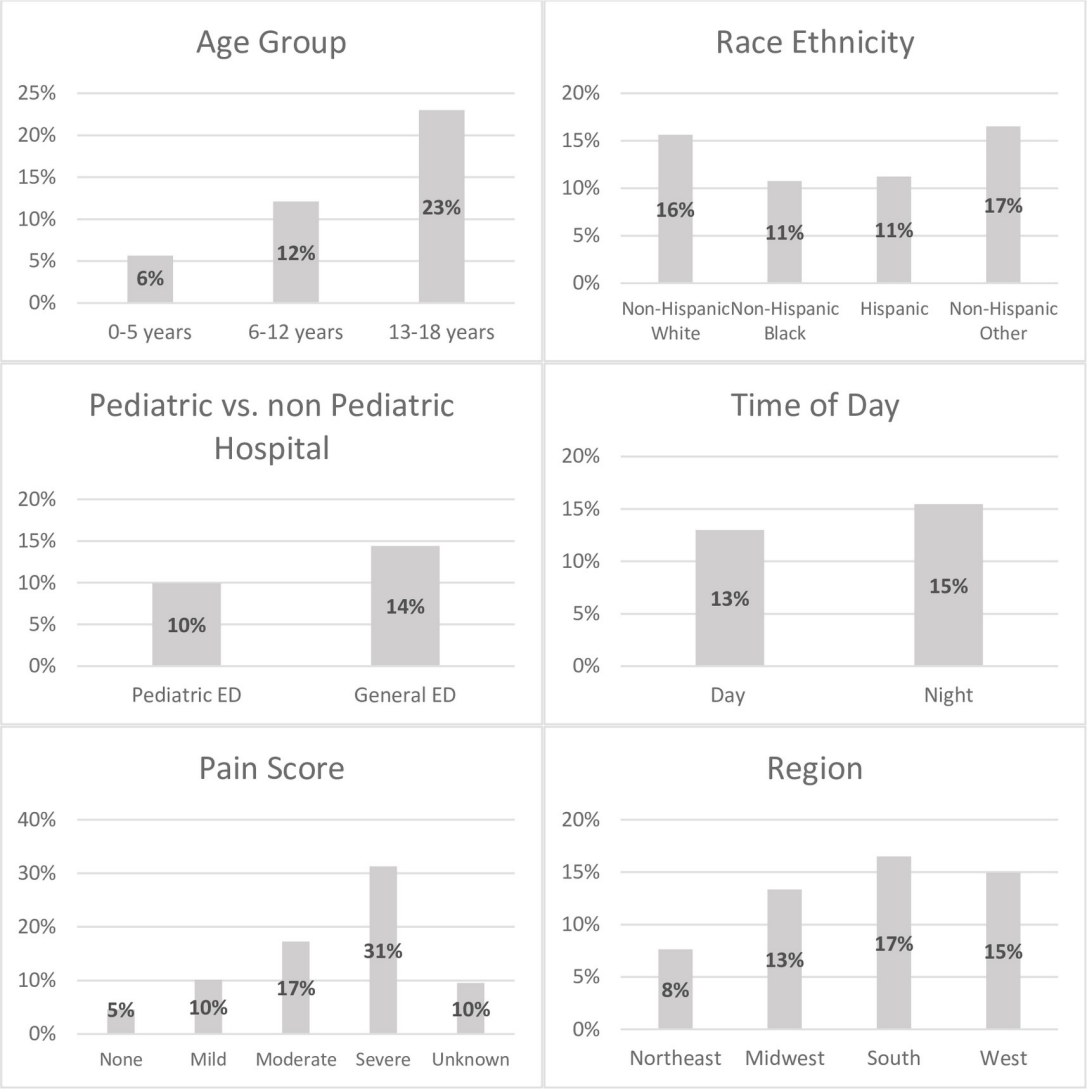
Proportion of all visits versus visits with opioids administered.

There was a higher proportion of visits associated with opioid administration seen in the adolescent age group 13–18 years old (23%) compared to children aged 6–12 years (12%) and 0–5 years (6%) (Fig 2). In univariable logistic regression analyses, ED visits associated with an opioid medication were more likely to be for patients who were 13–18 years old, had private insurance and resided in the South, West, or Midwest (Table 1). ED visits associated with opioid administration were more likely to occur in the evening. Approximately six percent (6%) of visits who received opioid medications had a pain score of “none” with increasing percentage of opioid administration as pain severity increased (p<0.001). For both visits that received an opioid and did not receive an opioid, the median of the injury severity score per visit was 3 (IQR 2,3). The proportion of visits with a severe injury score (4 and 5) was 6% for encounters that received opioids compared to 3% for those who did not receive opioids (p<0.001). Of the estimated 8.5 million visits seen in a pediatric ED, 10% received an opioid compared to non-pediatric EDs where 14% of visits received opioids. There was no difference in opioid administration rates in visits based on recent revisit.

**Table 1:** Demographic characteristics of ED patients who present with trauma diagnoses.

	All Visits		Given Opioid		No Opioid Given		P-Value
	n* = 81,226,000	%	n* = 11,312,000	%	n* = 69,915,000	%	
**Age group**							<0.001
0–5 years	25,964,000	32	1,464,000	13	24,500,000	35	
6–12 years	26,181,000	32	3,164,000	28	23,017,000	33	
13–18 years	29,081,000	36	6,684,000	59	22,397,000	32	
**Gender**							0.09
Female	33,741,000	42	4,463,000	39	29,278,000	42	
Male	47,486,000	58	6,849,000	61	40,637,000	58	
**Race Ethnicity**							<0.001
Non-Hispanic White	48,147,000	59	7,520,000	66	40,627,000	58	
Non-Hispanic Black	15,701,000	19	1,691,000	15	14,011,000	20	
Hispanic	14,566,000	18	1,646,000	15	12,930,000	19	
Non-Hispanic Other	2,812,000	4	464,000	4	2,348,000	3	
**Injury Mechanism**							0.49
Unintentional	69,546,000	86	9,819,000	87	59,727,000	85	
Intentional	675,000	0.8	98,000	0.9	577,000	0.8	
Intent Unknown	5,998,000	7	767,000	7	5,230,000	8	
NA	5,007,000	6	627,000	6	4,380,000	6	
**Pain Severity**							<0.001
None	14,325,000	18	655,000	6	13,670,000	20	
Mild	10,826,000	13	1,001,000	9	9,825,000	14	
Moderate	19,368,000	24	3,067,000	27	16,301,000	23	
Severe	15,988,000	20	4,611,000	41	11,376,000	16	
Unknown	20,719,000	25	1,978,000	17	18,742,000	27	
**Time of Day**							<0.001
Day (7am-7pm)	50,347,000	62	6,541,000	58	43,806,000	63	
Night(7pm-7am)	30,879,000	38	4,770,000	42	26108000	37	
**Weekend Visit**	24,165,000	30	3,404,000	30	20,761,000	30	
**Insurance status**							<0.001
Private	32,913,000	40	5,045,000	45	27,869,000	40	
Public	34,085,000	42	3,950,000	35	30,135,000	43	
Self	6,306,000	8	1,157,000	10	5,148,000	7	
Other/Unknown	7,922,000	10	1,159,000	10	6,763,000	10	
**Geographic region**							<0.001
Northeast	14,106,000	17	1,078,000	10	13,028,000	19	
Midwest	18,207,000	23	2,427,000	22	15,780,000	22	
South	32,249,000	40	5,333,000	47	27,017,000	39	
West	16,564,000	20	2,475,000	22	14,090,000	20	
**Pediatric ED**	8,520,000	11	846,000	8	7,673,000	11	0.003
**Academic ED**	6,936,000	9	781,000	7	6,155,000	9	0.02
**Resident Present**	6,041,000	7	846,000	8	5,195,000	7	0.94
**Nurse Practitioner Present**	6,036,000	7	708,000	6	5,329,000	8	0.08
**Physician’s Assistant Present**	11,497,000	14	1,580,000	14	9,917,000	14	0.85
**Severe Injury Severity Score**	2,832,000	4	631,000	6	2,201,000	3	<0.001

*n, survey weighted estimate

In multivariable analyses, patients who received opioids were more likely to be 13–18 years old, and less likely to be Hispanic or non-Hispanic black. Opioid administration was associated with evening visits, the Southern region of the United States, severe pain score and a severe injury severity score (4–5) (Table 2).

**Table 2:** Multivariate analysis.

Age	Odds Ratio
0–5 years	Referent
6–12 years	1.44 (1.17, 1.78)
13–18 years	2.75 (2.21, 3.42)
**Gender**	
Female	Referent
Male	1.19 (1.03, 1.37)
**Race Ethnicity**	
Non-Hispanic White	Referent
Non-Hispanic Black	0.69 (0.57, 0.84)
Hispanic	0.84 (0.69, 1.04)
Non-Hispanic Other	1.33 (0.90, 1.97)
**Pain Severity**	
None	Referent
Mild	1.79 (1.26, 2.55)
Moderate	3.15 (2.32, 4.29)
Severe	6.48 (4.72, 8.92)
**Severe Severity Score (4–5)**	2.19 (1.59, 3.00)
**Time of Day**	
Day (7am-7pm)	Referent
Night (7pm-7am)	1.26 (1.09, 1.46)
**Insurance status**	
Private	Referent
Public	0.77 (0.66, 0.89)
Self	1.15 (0.91, 1.45)
Other	1.16 (0.86, 1.57)
**Geographic region**	
Northeast	Referent
Midwest	2.14 (1.67, 2.76)
South	2.97 (2.35, 3.76)
West	2.23 (1.71, 2.90)
**Pediatric ED**	0.73 (0.52, 1.03)
**Academic ED**	1.09 (0.82, 1.46)
**Nurse Practitioner Present**	0.77 (0.56, 1.06)

The most common diagnoses for the overall population of pediatric patients presenting to the ED discharged with a trauma diagnosis included head injury (23%), face contusion (17%) and ankle sprain (16%). Ankle sprain (530,000 estimated visits) and clavicle fracture (364,000 estimated visits) were associated with the greatest overall number of opioid exposures (Table 3A) while fracture of the low radius and fracture of the unspecified radius had the greatest rate of opioid administration (71% and 63% respectively, Table 3B).

**Table 3:** (A) Diagnoses with greatest number of opioids administered. (B) Diagnoses with highest proportion of opioid administration.

ICD9 Code	Total Visits with ICD9 Code	Visits with an Opioid	%
(A)			
Ankle Sprain	3,826,000	530,000	14
Fracture of Clavicle	754,000	364,000	48
Fracture of Distal Radius	778,000	340,000	44
Face Contusion	4,191,000	283,000	7
Fracture of Carpal Bone	645,000	276,000	43
(B)			
Fracture of Low Radius	279,000	199,000	71
Fracture of Unspecified Radius	242,000	152,000	63
Fracture of Clavicle	754,000	364,000	48
Fracture of Ankle	543,000	256,000	47
Fracture of Distal Radius	778,000	340,000	44

The most commonly administered opioid medications were acetaminophen-hydrocodone (5,403,000 estimated visits, 48%), acetaminophen-codeine (3,168,000 estimated visits, 28%), morphine (1,627,000 estimated visits, 14%) and acetaminophen-oxycodone (846,000 estimated visits, 8%). In patients under 12 years of age, the most common medication administered in the ED and prescribed at the end of the visit was acetaminophen-codeine, (33% given in ED, 55% prescribed, 62% both administered and prescribed).

## Discussion

To our knowledge, this is the first study reporting frequency of opioid administration in the pediatric population discharged from the ED with a trauma diagnosis. Although we report a decrease in opioid administration over time in pediatric patients presenting with a trauma diagnosis discharged from the ED, we found that a meaningful number, an estimated 900,000 visits in 2015, are still receiving opioid medications. We demonstrate a temporal trend of decreased rates of opioid administration for patients discharged home from the ED. While decreasing trends of opioid administration are encouraging, more work needs to be done as opioids are associated with potential adverse side effects, abuse potential, poisonings, and intentional and unintentional injury deaths. [3,22] The side effects of opioids include respiratory depression, sedation, nausea, constipation, dizziness, physical dependence and tolerance.[23] Additionally, the adolescent and young adult age group are at risk for abuse of prescription medications.[1,4] A recent study found high rates of opioid prescriptions for adolescent and young adults in ambulatory and emergency department settings.[24] Our study extends this prior investigation and highlights specific injury types at high risk for opioid exposure in this vulnerable age group. Furthermore, despite a decrease in opioid use in the US, there is an increase in the rate of opioid poisonings, unintentional injury death and opioid-related suspected suicides among teenagers. [3,22] Given the concerning trends in adolescent opioid use and adolescent opioid related injuries, special attention should be paid to the delivery of opioids to this vulnerable population.

Previous studies have reported a wide variation in opioid prescribing patterns throughout the United States that cannot be explained by painful conditions alone.[25] Understanding these factors may be important for future efforts designed to curtail the use of opioids. Our study indicates adolescent age is associated with opioid administration. Multiple studies have previously highlighted that adolescents are more likely to receive opioid prescriptions compared to the younger pediatric age groups. [26] This may be due to older children having increased ability to verbalize pain compared to younger patients or providers feeling more comfortable with opioid administration in the adolescent age group compared to infants and toddler-aged children. [26] Additionally, we found patients who receive opioids are less likely to be Hispanic or non-Hispanic black. Prior studies have reported similar racial discrepancies in opioid administration for pediatric patients with various disease conditions. [27–30] Furthermore, we found several patient level and hospital level factors associated with opioid administration including time of day, pain severity, severe injury severity score and region of the country. These findings are consistent with prior literature that highlights the association of opioid prescriptions and ongoing opioid use with high initial pain scores and high injury severity scores. [31,32] Lastly, the Southern region was associated with an increased risk for administering opioids. Regional variation has been reported in previous studies with speculation that such variability is due to disparate practice patterns or prescription drug monitoring programs.[1,4,24,26,33]

Opioid medications may not be an optimal analgesic particularly when a painful condition is related to a musculoskeletal injury that can be discharged from the ED. Multiple studies have shown that acetaminophen or nonsteroidal anti-inflammatory medications are effective first line medications for musculoskeletal pain. Additionally, nonsteroidal anti-inflammatory medications perform equally as well as opioids in reducing musculoskeletal pain caused by fracture and postoperative orthopedic pain with less adverse effects.[7,9,34–36] Given that there are strong data suggesting that even short-term use of opioids may lead to a predisposition to opioid dependence, emergency providers should use caution with administering opioids for patients that may receive equal benefit from non-opioid analgesia.[34,37–39] The drivers of physician prescribing practices, particularly the choice of opioids versus nonsteroidal anti-inflammatory medications are not clear. Several studies have suggested individual, high intensity prescribers may be responsible for a large number of opioid exposures and these providers may be less likely to use alternative analgesics.[37] Limited data are available on provider decision-making processes regarding medication choice for acute pain in pediatric patients presenting to the emergency department.

Additionally, we found that an estimated 655,000 visits with a pain severity score of “none” were associated with an opioid medication. Unfortunately, NHAMCS does not allow for a comprehensive evaluation of the clinical circumstances that might result in opioid administration in the absence of documented pain. However, these findings suggest pain status may not be the sole driver of opioid prescribing patterns. Moreover, for pain not responsive to nonsteroidal anti-inflammatory medications alone, or for procedural analgesia, opioid-sparing analgesia such as ketamine may be a viable alternative.

In our study, acetaminophen-hydrocodone was most frequently used, with acetaminophen-codeine as the next most frequently used in this patient population (and the most frequently administered opioid in children less than 12 years of age). Although acetaminophen-hydrocodone has demonstrated efficacy as an analgesic with a good safety profile in pediatric patients, codeine is a dangerous medication that should not be used due to variable metabolism that may lead to fatal toxicity in children.[19,33,40] National guidelines have recommended against the use of codeine in children for indications of analgesia and cough suppression.[33] A surprisingly high frequency of codeine prescriptions to pediatric patients has previously been reported and there have been ongoing recommendations to find safer alternatives to codeine.[24,26] Our study also suggests that ongoing efforts are needed to continue to reduce codeine utilization, particularly in patients under 12 years of age.

Our study has several limitations. First, the NHAMCs dataset is comprised of calculated estimates and although great care is taken to collect and calculate the numbers, the estimates may not accurately represent practice throughout the country. Additionally, we are unable to discern the clinical indication for opioid administration from the NHAMCS dataset, including procedural sedation or analgesia. Though we limited our analyses to a low acuity population of children and adolescents discharged from the ED, the judicious use of opioids in this setting may have important clinical benefits such as adequate pain control and avoidance of costly hospitalizations. Third, we are not able to evaluate whether or not the ED prescriptions were filled, nor were we able to determine the amount or duration of opioid prescribed. Fourth, although no apparent effect on our results, the pain score is missing in approximately one quarter of all measurements, potentially introducing bias. Fourth, NHAMCS does not allow for individual level or longitudinal patient data to ascertain adverse outcomes associated with opioid exposure. Lastly, though we purposefully limited our analyses to a low acuity population of pediatric trauma patients discharged from the ED, it is possible that our data set included a more severe case mix.

In conclusion, despite an overall decreasing trend in opioid administration for pediatric trauma patients discharged from the ED, approximately one million children each year are exposed to opioids in this setting, often for conditions that have been found to potentially respond well to management with nonsteroidal anti-inflammatory medications alone. Codeine continues to be frequently administered, in spite of national guidelines recommending against its use. These findings could inform targeted interventions and educational programs designed to minimize exposure to opioids in children.
